# Nursing Leadership for 21st Century

**DOI:** 10.1590/1518-8345.0000.3472

**Published:** 2021-08-30

**Authors:** Robert L. Anders, Debra Jackson, Patricia M. Davidson, John P. Daly

**Affiliations:** 1 University of Texas, El Paso, TX, United States of America.; 2 University of Sydney, Susan Wakil School of Nursing, Sydney, NSW, Australia.; 3 Johns Hopkins University, School of Nursing, Baltimore, MD, United States of America.

**Figure:**
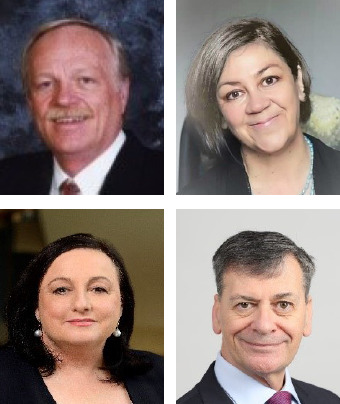

The COVID-19 pandemic has created both a crisis and an opportunity in nursing leadership. There are no parts of the world that have not been touched by the pandemic and undeniably this has placed significant stress on the nursing workforce. Nursing leadership is under tremendous stress to provide the staff and resources to care for what seems a relentless demand.

No longer can we continue to discuss the need for effective nursing leadership. The time to make changes is now. There must be a call to action to ensure nursing leaders at all levels of management have not only the competency and expertise to move and lead organizations. These leaders must have a seat at the table. Nursing leaders need to be able to not only create and participate in health policy decisions. They must also be able to manage healthcare team members throughout the healthcare organization. Of critical importance is the leadership provided to those nurses at the bedside.

Inconsistencies in the educational preparation of nurse leaders and lack of consistent training have, in part, impacted our responsibility to lead. The 21^st^ century and beyond means we must educate and mentor a new cadre of nurse leaders. In a recent published study it has been argued for nurses’ potential to assume a far more significant role in designing and developing health services. The time is ripe for a nurse-led and revolutionary change of focus to address long-term conditions, infectious and non-infectious diseases, and the "diseases of despair"^(1)^. Nursing leaders must envisage and articulate the vision to achieve these goals and then work together to create the preferred future. Leaders need the necessary competencies to ensure that the nursing workforce can position itself to address the vision.

There must be a paradigm shift in nursing leaders’ education and mentoring, with a radical re-visioning of the qualities and skills needed for nurse leaders to take nursing forward into the 22nd century and beyond. The shift is necessary because the rapid changes that we are witnessing within the healthcare environment will continue. Leaders must be able to drive a rapidly change process plan to ensure success effectively. Researchers^(2)^ described how healthcare workers react to change and found for many change creates psychological uncertainty as to what the proposed change means to them personally and their practice. The immediate reactions to change were categorized as either indifference or passive resistance to change. The response to change is a concern for nursing in all spheres and emphasizes the power of the status quo. However, participation in or support for reform occurred when the health care practitioners implemented the changes or when the changes included their constructive feedback. When changes are well-conceived and well communicated^(2)^, the chances of adoption are high. Educating nursing leaders on how to facilitate and adapt to a rapidly changing healthcare environment is essential. These leaders can then enable the change process with their staff and colleagues more effectively.

## Leadership in a New World Order

Although there is diverse and meaningful literature on leadership, there are some unique attributes to consider in developing nursing leaders. First, we must consider this within a social-ecological framework. Socio-ecological models increase the understanding of the dynamic interrelations among micro, meso, and macro factors. Leadership cannot be viewed in a vacuum – it must be considered within the social, political, cultural, and economic context within which it is enacted. COVID-19 has underscored that we cannot consider health care external to a broader societal context. The lessons have been brutal but a wake-up call to assess nurses' role and function in a global context^(3)^.

Nursing leaders have long struggled to cast off oppression and suppression of their value, particularly within the health care environment. Physicians historically have been men and women nurses. Nurses practiced in a situation in which they were often oppressed, undervalued, and their practice was restrictive. The medical model, combined with sexism and gender-based roles, has influenced many healthcare organizations' nursing leadership behaviors^(4)^. There are also hierarchical issues within our profession and our narrative have privileged the voices of the status quo and in some instances perpetuated structural racism^(5)^.

As health care complexity has increased, the need for effective, well prepared nursing leaders has never been more urgent. The practice of promoting the "great clinical nurses" into management is no longer a viable model. Healthcare organizations' success in meeting fiscal, patient satisfaction, and quality of care goals is mainly dependent on nursing leadership's leadership competencies. To promote competency in nursing leaders and to build infrastructures that encourage the creation of expertise, nurse leaders need to embrace evidence-based approaches^(6)^, as well as a range of other skills and qualities.

Nurse leadership in healthcare organizations is accountable and responsible for many patients' outcomes, including patient satisfaction. When nursing leaders have the authority and resources, they need to manage their respective units with accountability. Thus, when held accountable by senior management, the nurse leader needs autonomy and means to achieve the identified goals. Nurse leaders must be able to create and communicate a vision for their areas of responsibility. They need to build positive relations with those they lead and adopt plans and actions to achieve mutual objectives to be successful^(7)^.

In some employment settings, the nurse leader may need to leverage nursing's importance with senior management. The leverage is particularly essential when the nursing leader uses transformation leadership with an environment that appreciates nurses. Their contributions to patient outcomes are evident. Along with better patient outcomes can also come a more efficient fiscal operation. Thus, the nursing leader needs to ensure senior leadership "gets" the value of supporting nurses with the autonomy and resources to help the organization achieve its vision^(7)^. **Nursing Leadership during COVD-19**

COVID-19 creates the urgent need for nursing leaders who can advocate for their staff. There must be trust and confidence between the frontline nurses and nurse leaders. The communication must be frequent and continuous with a back-and-forth dialogue. Ensuring there are regular structured communication avenues such as shift huddles, so the staff has an awareness of employer action and updates related to COVID-19. Safeguarding staff so they are properly educated to manage COVID-19 patients, particularly having sufficient personal protective equipment and understand how to minimize exposure to the virus, is essential. The nursing leader also needs to facilitate staff having appropriate rest breaks while on duty. Given the high level of stress for both patients and staff, nursing leadership's www.eerp.usp.br/rlae

Anders RL, Jackson. D, Davidson PM, Daly JP. 3 attention to listen and be supportive of staff is critical. The establishment and maintenance of a caring relationship with the nursing staff cannot be understated.

Challenges to maintaining resilience in the face of the ongoing death of patients are difficult. Moral distress and burnout from prolonged exposure to COVID-19 patients and family are challenges nursing leadership needs to be aware of. Nursing leaders need awareness of their staff and understand what pro-active steps need to be taken to address such. Eventually, as the population becomes vaccinated and the demands of caring for COVID-19 patients decrease, nursing cannot go back to operating as before. Nursing leadership needs to learn from the current pandemic and apply these lessons moving forward to future proof our profession and future populations.
